# Genomic Update of Phenotypic Prediction Rule for Methicillin-Resistant Staphylococcus aureus (MRSA) USA300 Discloses Jail Transmission Networks with Increased Resistance

**DOI:** 10.1128/spectrum.00376-21

**Published:** 2021-07-21

**Authors:** Sarah E. Sansom, Emily Benedict, Stephanie N. Thiede, Bala Hota, Alla Aroutcheva, Darjai Payne, Chad Zawitz, Evan S. Snitkin, Stefan J. Green, Robert A. Weinstein, Kyle J. Popovich

**Affiliations:** a Division of Infectious Diseases, Rush University Medical Center /Cook County Health, Chicago, Illinois, USA; b Department of Microbiology and Immunology, University of Michigan, Ann Arbor, Michigan, USA; c Division of Infectious Diseases, Cook County Health/Cermak Health Services, Chicago, Illinois, USA; Memorial Sloan Kettering Cancer Center

**Keywords:** MRSA, *Staphylococcus aureus*, antibiotic resistance, methicillin resistance

## Abstract

Methicillin-resistant Staphylococcus aureus (MRSA) is a leading cause of health care-associated (HA) and community-associated (CA) infections. USA300 strains are historically CA-MRSA, while USA100 strains are HA-MRSA. Here, we update an antibiotic prediction rule to distinguish these two genotypes based on antibiotic resistance phenotype using whole-genome sequencing (WGS), a more discriminatory methodology than pulsed-field gel electrophoresis (PFGE). MRSA clinical isolates collected from 2007 to 2017 underwent WGS; associated epidemiologic data were ascertained. In developing the rule, we examined MRSA isolates that included a population with a history of incarceration. Performance characteristics of antibiotic susceptibility for predicting USA300 compared to USA100, as defined by WGS, were examined. Phylogenetic analysis was performed to examine resistant USA300 clades. We identified 275 isolates (221 USA300, 54 USA100). Combination susceptibility to clindamycin or levofloxacin performed the best overall (sensitivity 80.7%, specificity 75.9%) to identify USA300. The average number of antibiotic classes with resistance was higher for USA100 (3 versus 2, *P* < 0.001). Resistance to ≤2 classes was predictive for USA300 (area under the curve (AUC) 0.84, 95% confidence interval 0.78 to 0.90). Phylogenetic analysis identified a cluster of USA300 strains characterized by increased resistance among incarcerated individuals. Using a combination of clindamycin or levofloxacin susceptibility, or resistance to ≤2 antibiotic classes, was predictive of USA300 as defined by WGS. Increased resistance was observed among individuals with incarceration exposure, suggesting circulation of a more resistant USA300 clade among at-risk community networks. Our phenotypic prediction rule could be used as an epidemiologic tool to describe community and nosocomial shifts in USA300 MRSA and quickly identify emergence of lineages with increased resistance.

**IMPORTANCE** Methicillin-resistant Staphylococcus aureus (MRSA) is an important cause of health care-associated (HA) and community-associated (CA) infections, but the epidemiology of these strains (USA100 and USA300, respectively) now overlaps in health care settings. Although sequencing technology has become more available, many health care facilities still lack the capabilities to perform these analyses. In this study, we update a simple prediction rule based on antibiotic resistance phenotype with integration of whole-genome sequencing (WGS) to predict strain type based on antibiotic resistance profiles that can be used in settings without access to molecular strain typing methods. This prediction rule has many potential epidemiologic applications, such as analysis of retrospective data sets, regional monitoring, and ongoing surveillance of CA-MRSA infection trends. We demonstrate application of this rule to identify an emerging USA300 strain with increased antibiotic resistance among incarcerated individuals that deviates from the rule.

## INTRODUCTION

Methicillin-resistant Staphylococcus aureus (MRSA) has been an increasingly important cause of both health care-associated (HA) and community-associated (CA) infections over the past several decades (1–4). CA-MRSA is increasingly recognized as an important cause of nosocomial infections (5–7). By pulsed-field gel electrophoresis (PFGE), USA300 has been identified as the most common strain of CA-MRSA and can lead to severe infections in previously healthy individuals (8–10). USA100 is a strain of MRSA traditionally associated with health care exposures and remains an important cause of bloodstream infection in hospitals (11). While HA-MRSA infection rates have declined (12), the incidence of invasive CA-MRSA infections remains largely unchanged (13–15). As USA300 has emerged as an important cause of hospital-onset MRSA infections (5, 16–18), the ability to differentiate between USA300 and USA100 can have useful epidemiologic and treatment implications.

Historically, USA300 strains have increased susceptibility to non-β-lactam antibiotics. By contrast, USA100 strains often display resistance to multiple antibiotic classes (19). Clindamycin and levofloxacin resistance have been suggested as potentially useful phenotypic markers to distinguish between the USA100 and USA300 strains (20). Preserved susceptibility to trimethoprim-sulfamethoxazole and tetracyclines has been described at greater than 90% prevalence in CA-MRSA skin and soft tissue infections (21). However, multidrug-resistant (MDR) USA300 MRSA is emerging as a community-associated infection in some high-risk populations, including men who have sex with men (22).

While PFGE has traditionally been used to distinguish strains of MRSA, it has limited ability to differentiate between endemic MRSA strains. In addition, studies using WGS have shown that PFGE can misclassify strains (23, 24). WGS resolution is superior to PFGE and is now considered the gold standard for MRSA strain typing (25, 26). Our previous work (27) used performance characteristics of the antimicrobial susceptibility phenotype, a “phenotypic rule”, to predict genotype as identified by PFGE. Our aim is to update this phenotypic prediction rule for distinguishing between common MRSA genotypes using WGS and current antibiotic resistance patterns. We examined performance of antimicrobial susceptibility and number of resistant antibiotics as a tool to distinguish between USA300 and USA100 in clinical MRSA isolates. This rule is intended to be used for discrimination of USA300 in both community and hospital isolates to study the evolving epidemiology of MRSA. We also demonstrate how our approach disclosed possible networks of MRSA transmission based on deviations from the established rule in a population with a history of incarceration.

## RESULTS

MRSA isolates (n = 275) were included in the analysis, with 221 isolates identified as USA300 and 54 isolates identified as USA100. Performance characteristics for predicting genotype, for selected individual, and for combinations of antibiotics are listed in Table 1. Susceptibility to clindamycin (sensitivity [Sn] 63.3%, specificity [Sp] 88.9%, likelihood ratio [LR] 5.7 [95% confidence interval (CI) 2.7 to 12.2]) performed the best for an individual antibiotic to predict USA300, followed by levofloxacin (Sn 62.4%, Sp 81.5%, LR 3.4 [95% CI 1.9 to 6.0]). Tetracycline, gentamicin, and rifampin had high sensitivity but poor specificity for predicting USA300 (Sn 99.1%, 100%, and 99.1%, respectively; Sp 18.5%, 7.4%, and 5.6%, respectively). Combination of susceptibility to clindamycin or levofloxacin performed the best overall to predict USA300 (Sn 80.7%, Sp 75.9%, LR 3.4 [95% CI 2.1 to 5.4]). Combination of susceptibility to both clindamycin and levofloxacin had excellent specificity but poor sensitivity (Sn 45.1%, Sp 94.4%, LR 8.2 [95% CI 2.7 to 24.8]).

**TABLE 1:** Antibiotic susceptibility phenotype: performance characteristics to predict USA300

Susceptible to antibiotic^*a*^	Sensitivity (%)	Specificity (%)	Positive LR (95% CI)	Prevalence, 25%		Prevalence, 50%		Prevalence, 75%	
				PPV (%)	NPV (%)	PPV (%)	NPV (%)	PPV (%)	NPV (%)
Levofloxacin and clindamycin	45.4	94.4	8.2 (2.7–24.8)	73.2	83.9	89.1	63.4	96.1	36.6
Clindamycin and tetracycline	62.8	90.7	6.8 (2.9–15.7)	69.4	88.0	87.2	71.0	95.3	44.9
**Clindamycin** ^*b*^	**63.3**	**88.9**	**5.7** **(2.7–12.2)**	**65.5**	**87.9**	**85.1**	**70.8**	**94.5**	**44.7**
**Levofloxacin**	**62.4**	**81.5**	**3.4** **(1.9–6.0)**	**53.0**	**86.7**	**77.1**	**68.5**	**91.0**	**42.0**
**Levofloxacin or clindamycin**	**80.7**	**75.9**	**3.4** **(2.1–5.4)**	**52.8**	**92.2**	**77.0**	**79.8**	**91.0**	**56.8**
Tetracycline	99.1	18.5	1.2 (1.1–1.4)	28.9	98.4	54.9	95.3	78.5	87.2
Gentamicin	100	7.4	1.1 (1.0–1.2)	26.5	100	51.9	100	76.4	100
Rifampin	99.1	5.6	1.1 (1.0–1.1)	25.9	94.9	51.2	86.0	75.9	67.2

a Antibiotics excluded due to resistance >90% included erythromycin; antibiotics excluded due to susceptibility >99% included trimethoprim-sulfamethoxazole, linezolid, Synercid, and vancomycin.

b Bolded text indicates the most discriminatory antibiotic(s) to predict USA300 MRSA.

The positive predictive value (PPV) of each rule for identifying USA300 strains increased with higher prevalence. When the prevalence of USA300 strains was 25%, 50%, and 75%, the PPVs for identifying USA300 were 65.5%, 85.1%, and 94.5% for the clindamycin-susceptible rule and 53.0%, 77.1%, and 91.0% for the levofloxacin-susceptible rule, respectively. The PPVs for identifying USA300 strains for the combined clindamycin or levofloxacin-susceptible rule were 52.8%, 77.0%, and 91.0% when the prevalence of USA300 was 25%, 50%, and 75%, respectively.

**FIG 1:**
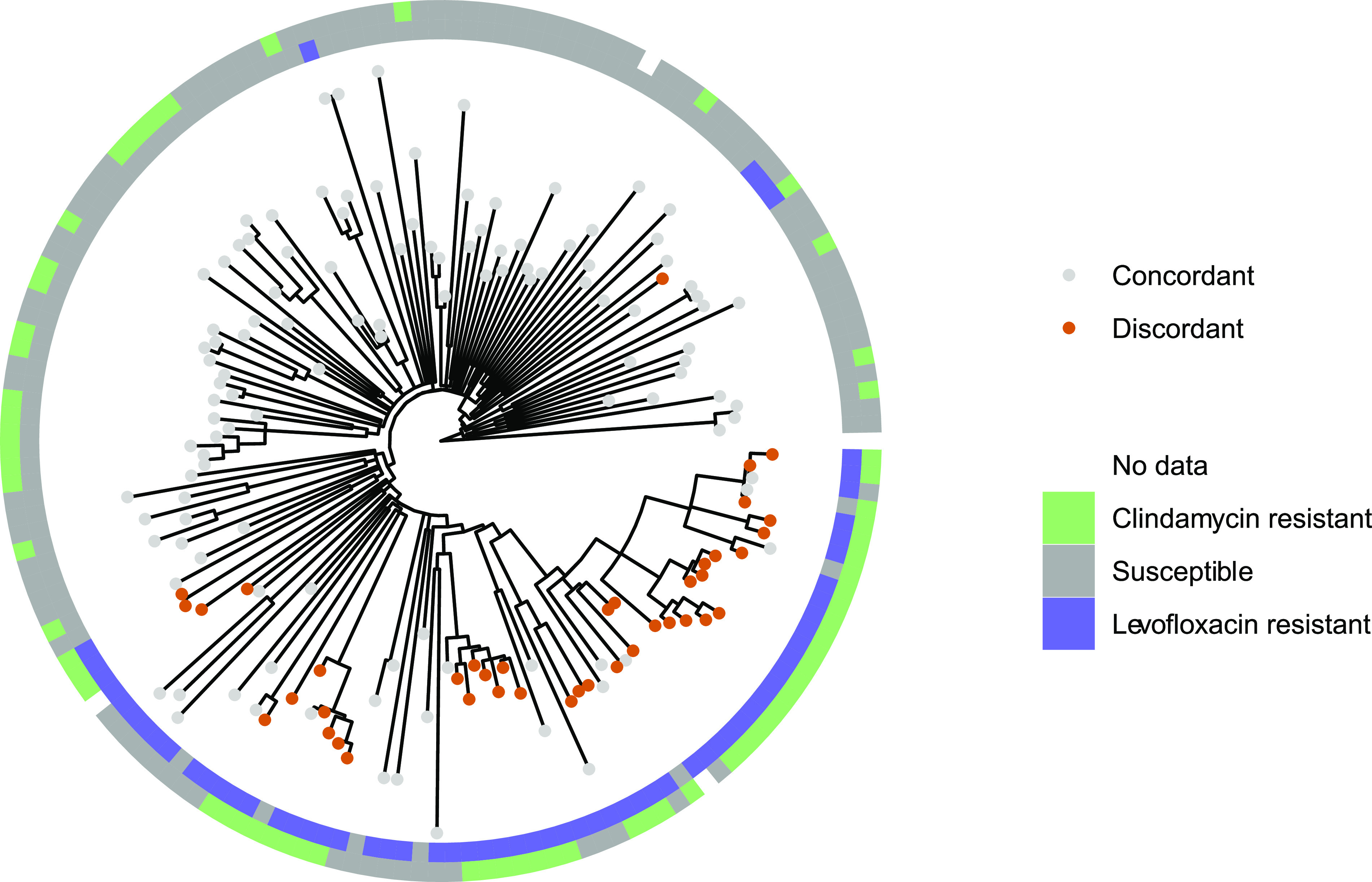
Phylogenetic tree of USA300 incarceration isolates annotated with prediction concordance or discordance. A recombination-masked whole-genome alignment was used to make a maximum likelihood phylogeny of USA300 clinical isolates from incarcerated individuals. The tree is midpoint rooted and annotated with resistance or susceptibility to clindamycin and levofloxacin. Tree tips are labeled with prediction discordance (resistance to both clindamycin and levofloxacin) or prediction concordance (susceptibility to clindamycin, levofloxacin, or both). Clustering of isolates discordant with the prediction rule is observed, indicating circulation of dual clindamycin-levofloxacin-resistant USA300.

The Matthews correlation coefficient (MCC) for USA300 identification by the combined clindamycin or levofloxacin-susceptible rule, which inherently considers the dominance of USA300 in our data, is fair for all isolates (0.393) but strong for bloodstream-only isolates (0.754). Upon closer evaluation, the improved performance of the prediction rule in bloodstream-only isolates appears to be related to a group of USA300 isolates collected from the jail that were resistant to both clindamycin and levofloxacin. Phylogenetic analysis of USA300 jail isolates revealed a cluster characterized by resistance to both antibiotics (Fig. 1).

**FIG 2:**
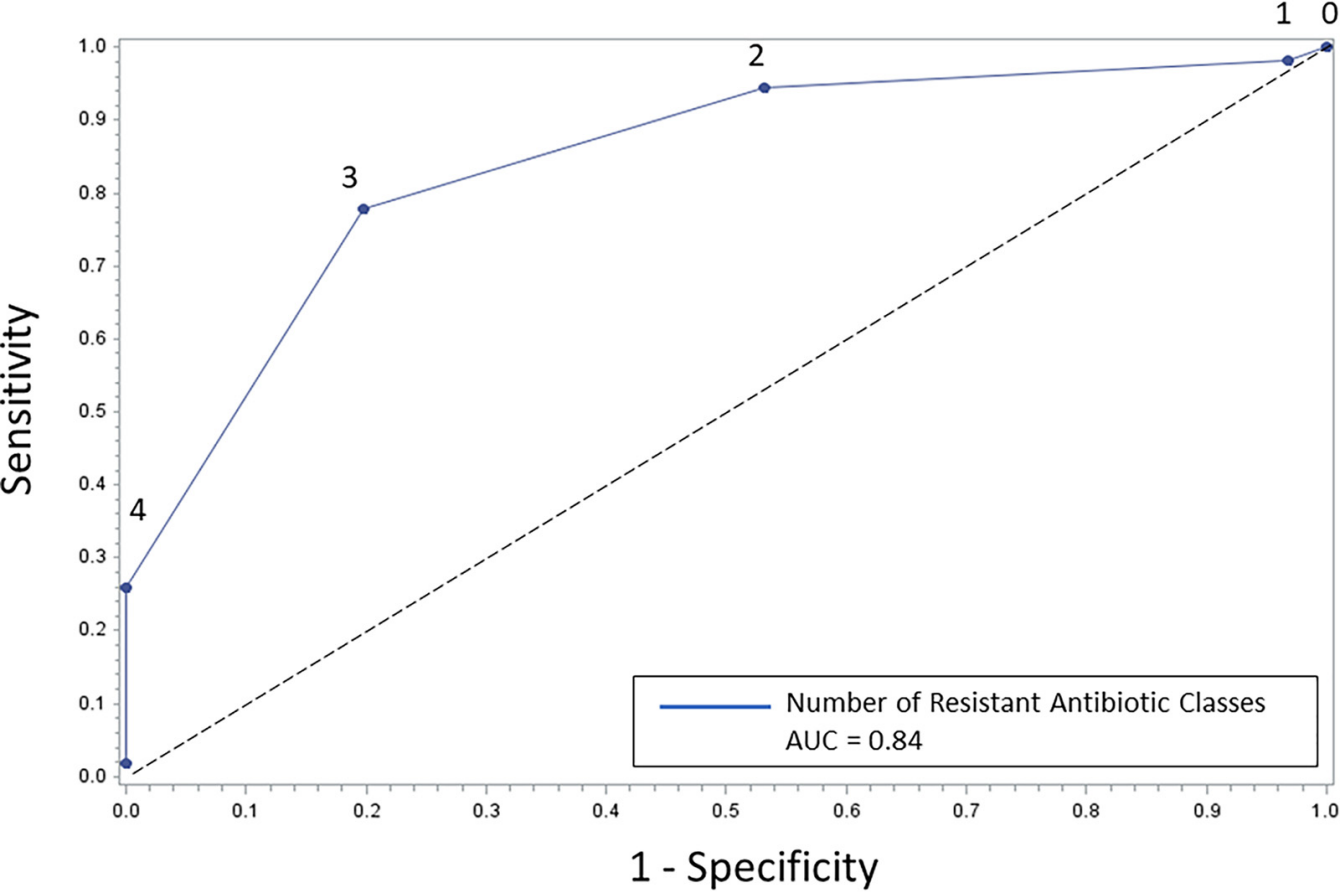
Number of resistant antibiotic classes as a predictor of USA300 MRSA. The numbers above the curve refer to the number of antibiotic classes to which the isolates expressed resistance. A cutoff point of resistance to ≤2 classes for USA300 and to ≥3 classes for USA100 isolates is seen (AUC 0.84, 95% CI 0.78 to 0.90). The cutoff point was calculated using the Youden index (48).

In a second analysis, we assessed resistance to antibiotics from six different classes. The average number of resistant antibiotic classes was higher for USA100 than for USA300 (3 versus 2, *P* < 0.001). The majority of USA300 isolates were resistant to ≤2 antibiotic classes (80.3%), with no USA300 isolates resistant to >3 antibiotics. The majority of USA100 isolates were resistant to ≥3 classes (77.8%). A cutoff of resistance to less than 3 antibiotic classes had the best diagnostic characteristics on a generated receiver-operator curve (ROC) (sensitivity 80.3%, specificity 77.8%, LR 3.6 [95% CI 2.2 to 6.0]; area under the curve [AUC] 0.84 [95% CI 0.78 to 0.90]). (Fig. 2) This suggests an optimal cutoff point of resistance to ≤2 classes to predict USA300 and ≥3 classes to predict USA100.

## DISCUSSION

Molecular subtyping methods, such as PFGE, do not sufficiently differentiate endemic MRSA strain types; therefore, use of these methods has previously limited our ability to understand transmission patterns of MRSA (24, 26). WGS can detect genetic differences between MRSA strains that are unidentifiable by other molecular epidemiologic methods and more accurately categorize MRSA strain types (26, 28). In this study, we evaluated antibiotic resistance phenotype as a predictor of genotype for WGS-defined USA300 strains from clinical MRSA isolates. We found that clindamycin and levofloxacin susceptibility were the best individual antibiotics for prediction of USA300, with the best overall performance seen with combined susceptibility to clindamycin or levofloxacin. The number of resistant antibiotics was useful, with a cutoff of ≤2 resistant antibiotic classes being predictive of USA300. Importantly, our analysis also identified a novel cluster of USA300 MRSA strains with increased resistance among individuals with incarceration history.

In comparison to our previously developed MRSA phenotypic prediction rule (27), clindamycin and fluoroquinolone susceptibility was found to be less sensitive and specific for prediction of CA-MRSA. In the current study, we used genomic sequencing to categorize MRSA strains as USA300 or USA100 and did not rely on PFGE as in our prior study (27), thus improving the accuracy of our derived rules. Additionally, the prior analysis utilized combinations of HA-MRSA (i.e., USA100, USA500, and USA800) and CA-MRSA (USA300 and USA400) isolates instead of limiting to the most common strain types (USA100 and USA300). Another potential explanation for these observed changes in the rule performance could be increasing prevalence of CA-MRSA antibiotic resistance. Increasing rates of clindamycin resistance among USA300 strains have been described (29), and regional variation exists for USA300 susceptibility patterns (22, 30). Although there are variations across the United States in rates of resistance of USA300 MRSA isolates to clindamycin, there are several studies that show nonsignificant trends that clindamycin may be better for reduction of recurrence and may be favored in some subgroups, such as children (31–33). Levofloxacin is not an agent that is typically used for the treatment of MRSA, but it remains clinically relevant for epidemiologic analyses. Our results support the importance of routinely updating antibiograms for MRSA isolates in both community and acute care settings. Clinicians should continue to submit isolates for culture and antimicrobial susceptibility to ensure continued monitoring for emergence of resistant strains and to inform epidemiologic analyses.

Previous attempts to classify MRSA as CA and HA strains included important clinical differences, such as antimicrobial resistance (24). However, exceptions to these rules have become more common (6, 22, 34). Additionally, as USA300 becomes a more ubiquitous cause of HA-MRSA infections, it has become less clear where acquisition of strains may be occurring (6, 19). Beyond the increased granularity of strain typing enabled by WGS, the inclusion of fully sequenced isolates in our study allowed phylogenetic analysis that revealed a closely related subset of USA300 jail isolates with increased antimicrobial resistance. This finding highlights the added strength of this phenotypic rule in that deviations from the rule may be useful to identify emergence of new resistant strains. This strain is unique from MDR USA300 clusters described in other cities, including San Francisco, California (22), and Austin, Texas (34). The prevalence of MRSA colonization at jail intake at our facility has been observed at nearly 1 in 5 inmates, with colonization associated with high-risk exposures in the community (i.e., injection drug use, HIV infection, unstable housing, and methamphetamine use) (35). High rates of recidivism and prior demonstration of MRSA community transmission networks among detainees (35) suggests that this more resistant USA300 MRSA cluster could have far-reaching implications for the treatment of MRSA infection in both the incarcerated population and associated at-risk community networks (i.e., where these individuals live).

A limitation of this study is that we included only the two most common MRSA clinical isolates (USA300, USA100) for analysis; other circulating MRSA strains (e.g., USA500 and USA800) were not included as they are less commonly isolated. So, it is unclear how the phenotypic prediction rule would perform when applied to non-USA300/non-USA100 MRSA strains. Additionally, this analysis was focused regionally and included fewer USA100 isolates than USA300 isolates. It may be useful for a community or health care setting to be familiar with circulating MRSA clones; our methodology could be adapted to a setting with differing prevalence of various MRSA strain types. Another limitation is that samples were collected from clinical isolates over time and likely reflect secular changes. Finally, the phenotypes described here reflect local antimicrobial resistance patterns (i.e., our local antibiogram) and may not be generalizable to all clinical settings. Differences in regional antimicrobial resistance patterns should be considered when applying these suggested phenotypic rules. A predictive phenotypic rule can easily be updated to account for changing antimicrobial resistance patterns. For example, to adapt this rule to a new region, researchers would submit a representative sample of clinical MRSA isolates for strain typing followed by analysis of antimicrobial resistance patterns. Similarly, changes in the antibiogram for MRSA, such as increasing antimicrobial resistance, would prompt the application of the same methodology to update the predictive phenotypic rule.

A phenotypic rule based on commonly available clinical information to predict genotype may be useful for epidemiologic applications, especially in the analysis of retrospective data or in a setting where strain typing is not available. It is reassuring that the prior PFGE-derived phenotypic rule has changed little with updates in the WGS strain typing methods among bloodstream isolates. However, a cluster of more resistant USA300 MRSA isolates among individuals with incarceration exposures suggests that phenotypic rules should be interpreted carefully, as local epidemiology is key for accuracy. As multidrug resistance becomes an increasing challenge and emerges in the community, this rule can be adapted to accommodate these shifts.

In this study, we updated a previously derived phenotypic prediction rule for USA300 by using WGS. Clindamycin and levofloxacin susceptibility were the best individual predictors for USA300, with the best overall performance seen with a two-antibiotic prediction rule with susceptibility to clindamycin or levofloxacin. However, such phenotypic rules should be applied and interpreted in the context of local MRSA epidemiology. Emergence of more resistant USA300 MRSA among individuals with exposure to incarceration has important treatment and infection control implications for at-risk community networks.

## MATERIALS AND METHODS

### Study design.

Cook County Health (CCH) is the major safety net health care network in Cook County and has a comprehensive, integrated system of health care throughout Chicago, Illinois, and suburban Cook County. Included in this system are hospitals (including Stroger Hospital of Cook County), ambulatory and community health network clinics, a correctional health care facility, and an outpatient infectious disease clinic. We utilized a deduplicated convenience sample of existing MRSA clinical isolates from 2007 to 2017 cultured from individuals 18 years of age and older who received care at CCH. Clinical isolates were primarily from skin and soft tissue infections and bloodstream infections, but body fluid, urine, and respiratory tract isolates were also included. Nares surveillance cultures were excluded. Isolates were selected based on previously identified strain type using WGS. Antibiotic susceptibility was determined by the clinical microbiology laboratory with a MicroScan WalkAway 96 plus (Brea, CA). The study was approved with waiver of consent by the CCH institutional review board.

### Genomic analysis.

We utilized MRSA isolates related to USA300 or USA100 that had already undergone genomic sequencing as well as sequenced additional historical PFGE-predicted isolates to enrich the sample with USA100 strains. A total of 170 previously sequenced isolates from the Cook County Jail (8 USA100, 162 USA300), predominantly from skin and soft tissue infections (BioProject PRJNA638400) (35), as well as 71 previously sequenced isolates from MRSA bacteremia at Cook County Hospital (17 USA100, 54 USA300) were included (BioProject PRJNA345238) (6). Due to the low number of available previously sequenced USA100 isolates, an additional 34 contemporaneous PFGE-predicted USA100 bacteremia isolates at Cook County Hospital were submitted for sequencing (29 USA100, 5 USA300). Genomic DNA extracted from MRSA isolates submitted under BioProject PRJNA744410 was prepared for sequencing using a Nextera XT library preparation kit (Illumina, San Diego, CA) according to the manufacturer’s instructions. Sequencing was performed on an Illumina NextSeq500 instrument using a high-output kit with paired-end 2 × 150 bp reads. Library preparation and sequencing were performed at the University of Illinois Chicago Sequencing Core (UICSQC). Details on sequenced strains are available in Table S1 in the supplemental material. The quality of sequencing reads was assessed with FastQC (36), and Trimmomatic (37) was used for trimming adapter sequences and low-quality bases.

### Phylogenetic analysis and *in silico* PFGE classification.

Results of our revised prediction rule suggested existence of a more resistant strain population that we studied with additional phylogenetic analyses. Single nucleotide variants (SNVs) were identified by mapping cleaned sequencing reads against a finished USA300 MRSA reference genome and applying a series of filters to retain high quality variants. The variant calling pipeline can be found on GitHub at https://github.com/Snitkin-Lab-Umich/variant_calling_pipeline. After generating the whole-genome alignment for the isolates from the Cook County jail (PRJNA638400), we then masked sites identified as recombinant by Gubbins (38) and used this masked whole-genome alignment to build a maximum likelihood phylogeny with IQ-TREE version 1.6.12 (39) using the ModelFinder option to choose the best fit substitution model and ultrafast bootstrap with 1,000 replicates (-bb 1000) (40). The tree was midpoint rooted using the midpoint.root function in phytools (41). Non-USA300 tips were dropped using the drop.tip function in ape (42). Resistance data were overlaid on the tree with ggtree (43).

We classified isolates as USA300 using sequence probes provided by Bowers et al. (44) that included the probes innerCC8, CC8f, and CC8e or CC8e and CC8f. Isolates underwent multilocus sequence typing with ARIBA (45). Isolates that were not part of CC8 were further investigated for relatedness to USA100. Two USA100 genomes and a USA800 genome (USA100-NRS642, USA100-NRS720, USA800-NRS387 from BioProject PRJNA374337) and non-CC8 genomes from our study were assembled with an internal pipeline available at https://github.com/Snitkin-Lab-Umich/assemblage and aligned with cognac (46), and a phylogenetic tree was created with fasttree (47). Isolates with ST5 or that clustered with the USA100 reference genomes were considered USA100 (Fig. S1).

### Statistical analysis.

Analysis was performed using SAS software (version 9.4, SAS Institute, Cary, NC), Python (version 3.7, Python Software Foundation, available at http://www.python.org), and R (R version 4.0.3, R Foundation for Statistical Computing, Vienna, Austria.). The sensitivity (Sn), specificity (Sp), positive and negative predictive value (PPV and NPV), and likelihood ratios (LRs) were calculated for each unique antibiotic phenotype and antibiotic combinations. For these analyses, “true positive” was defined as USA300 that was susceptible to the antibiotic(s), “false negative” was defined as USA300 that was resistant to the antibiotic(s), “false positive” was defined as USA100 that was susceptible to the antibiotic(s), and “true negative” was defined as USA100 that was resistant to the antibiotic(s). The Matthews correlation coefficient (MCC) was calculated to assess performance of the highest-performing predictor to determine its predictive performance while accounting for unbalanced data sets. Antibiotics with very high levels of resistance (>90%; erythromycin) or susceptibility (>99%; trimethoprim-sulfamethoxazole, linezolid, Synercid, and vancomycin) were excluded from this analysis because they were unlikely to be discriminatory. The antibiotic phenotype for each unique isolate was then used to calculate the number of resistant antibiotic classes (using tetracycline, levofloxacin, rifampin, gentamicin, erythromycin, and clindamycin), from which a receiver-operator curve (ROC) was constructed.

### Data availability.

Sequencing data sets are available and can be found at BioProject PRJNA638400 (35), PRJNA345238 (6), and BioProject PRJNA744410.
