# Supplementary figures and images for: Genomic Update of Phenotypic Prediction Rule for Methicillin-Resistant Staphylococcus aureus (MRSA) USA300 Discloses Jail Transmission Networks with Increased Resistance
Source: Microbiol Spectr. 2021 Jul 21;9(1):10.1128/spectrum.00376-21. doi: 10.1128/spectrum.00376-21 (PMC8552710; doi:10.1128/spectrum.00376-21)

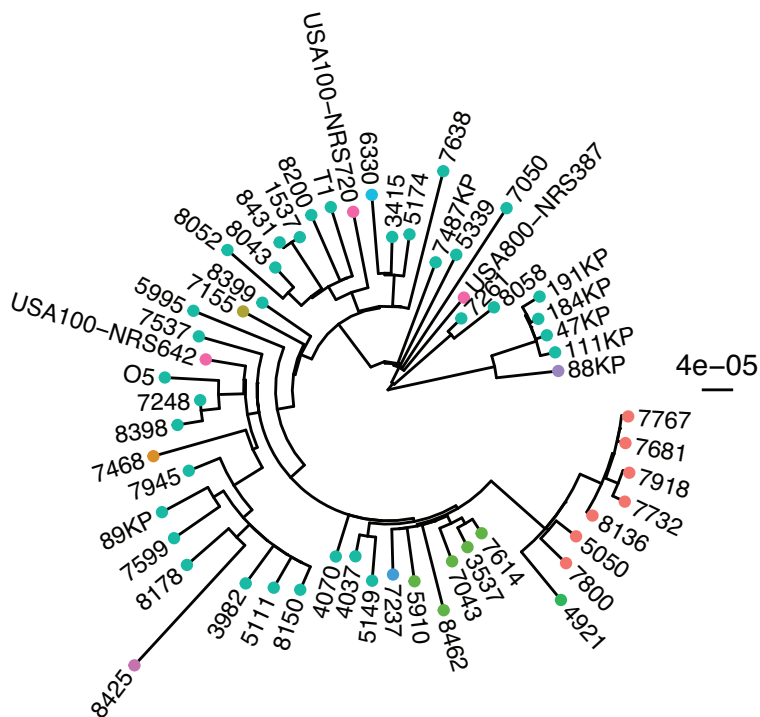

## MLST

- 105
- 1730
- 225
- 231
- 3390
- 5
- 5\*
- 809
- 840
- Novel\*
- Reference

Supplement: SUPPLEMENTAL FILE 2 — Supplemental material. Download SPECTRUM00376-21_Supp_2_seq4.pdf, PDF file, 0.03 MB. [file spectrum00376-21_supp_2_seq4.pdf]
