# Supplementary material for: Genomic Update of Phenotypic Prediction Rule for Methicillin-Resistant Staphylococcus aureus (MRSA) USA300 Discloses Jail Transmission Networks with Increased Resistance
Source: Microbiol Spectr. 2021 Jul 21;9(1):10.1128/spectrum.00376-21. doi: 10.1128/spectrum.00376-21 (PMC8552710; doi:10.1128/spectrum.00376-21)
Supplement: SUPPLEMENTAL FILE 3 — Supplemental material. Download SPECTRUM00376-21_Supp_3_seq8.pdf, PDF file, 0.1 MB. [file spectrum00376-21_supp_3_seq8.pdf]

Supplemental Material Legend

Supplemental Figure S1. USA100 classification using cognac tree. Sequences used in this study and three publicly available reference genomes were assembled using an internal assembly pipeline that can be found on Github: https://github.com/Snitkin-Lab-Umich/assembly_umich. After assemblies were aligned with cognac (1), a maximum likelihood phylogeny was made with FastTree (2). Scale bar indicates substitutions per site. MLST was generated by ARIBA (3) and overlayed on the tree with ggtree (4). For the purposes of this analysis, all isolates on this tree were considered USA100 on the basis that they clustered with either of the 2 USA100 reference genomes or were ST5.

REFERENCES

1. Crawford RD, Snitkin ES. cognac: rapid generation of concatenated gene alignments for phylogenetic inference from large, bacterial whole genome sequencing datasets. BMC Bioinformatics. 2021 Feb 15;22(1):70.

2. Price MN, Dehal PS, Arkin AP. FastTree 2 – Approximately Maximum-Likelihood Trees for Large Alignments. PLoS ONE [Internet]. 2010 Mar 10 [cited 2021 Feb 18];5(3). Available from: https://www.ncbi.nlm.nih.gov/pmc/articles/PMC2835736/ PMCID: PMC2835736

3. Hunt M, Mather AE, Sánchez-Busó L, Page AJ, Parkhill J, Keane JA, Harris SR. ARIBA: rapid antimicrobial resistance genotyping directly from sequencing reads. Microb Genomics [Internet]. 2017 Sep 4 [cited 2020 Jun 18];3(10). Available from: https://www.ncbi.nlm.nih.gov/pmc/articles/PMC5695208/ PMCID: PMC5695208

4. Yu G, Smith DK, Zhu H, Guan Y, Lam TT-Y. ggtree: an r package for visualization and annotation of phylogenetic trees with their covariates and other associated data. Methods Ecol Evol. 2017;8(1):28–36.

Supplemental Dataset S1. Analytic dataset of MRSA isolates used for analysis. Information includes strain type by whole genome sequencing (USA300 or USA100) and predicted strain type based on phenotypic prediction rule. MLST designations are also included.
